# Consideration of Sex, Gender, or Age on Outcomes of Digital Technologies for Treatment and Monitoring of Chronic Obstructive Pulmonary Disease: Overview of Systematic Reviews

**DOI:** 10.2196/49639

**Published:** 2023-11-29

**Authors:** Katja Matthias, Ivonne Honekamp, Monique Heinrich, Karina Karolina De Santis

**Affiliations:** 1 Faculty of Electrical Engineering and Computer Science University of Applied Science Stralsund Stralsund Germany; 2 Faculty of Business University of Applied Science Stralsund Stralsund Germany; 3 Department of Prevention and Evaluation Leibniz Institute for Prevention Research and Epidemiology - BIPS Bremen Germany

**Keywords:** digital technologies, digital intervention, COPD, AMSTAR 2, chronic obstructive pulmonary disease, gender, sex, age, overview, systematic review, treatment, monitoring, chronic disease, chronic illness, outcome reporting, review methodology, critical appraisal

## Abstract

**Background:**

Several systematic reviews have addressed digital technology use for treatment and monitoring of chronic obstructive pulmonary disease (COPD).

**Objective:**

This study aimed to assess if systematic reviews considered the effects of sex, gender, or age on the outcomes of digital technologies for treatment and monitoring of COPD through an overview of such systematic reviews. The objectives of this overview were to (1) describe the definitions of sex or gender used in reviews; (2) determine whether the consideration of sex, gender, or age was planned in reviews; (3) determine whether sex, gender, or age was reported in review results; (4) determine whether sex, gender, or age was incorporated in implications for clinical practice in reviews; and (5) create an evidence map for development of individualized clinical recommendations for COPD based on sex, gender, or age diversity.

**Methods:**

MEDLINE, the Cochrane Library, Epistemonikos, Web of Science, and the bibliographies of the included systematic reviews were searched to June 2022. Inclusion was based on the PICOS framework: (1) population (COPD), (2) intervention (any digital technology), (3) comparison (any), (4) outcome (any), and (5) study type (systematic review). Studies were independently selected by 2 authors based on title and abstract and full-text screening. Data were extracted by 1 author and checked by another author. Data items included systematic review characteristics; PICOS criteria; and variables related to sex, gender, or age. Systematic reviews were appraised using A Measurement Tool to Assess Systematic Reviews, version 2 (AMSTAR 2). Data were synthesized using descriptive statistics.

**Results:**

Of 1439 records, 30 systematic reviews published between 2010 and 2022 were included in this overview. The confidence in the results of 25 of the 30 (83%) reviews was critically low according to AMSTAR 2. The reviews focused on user outcomes that potentially depend on sex, gender, or age, such as efficacy or effectiveness (25/30, 83%) and acceptance, satisfaction, or adherence (3/30, 10%) to digital technologies for COPD. Reviews reported sex or gender (19/30 systematic reviews) or age (25/30 systematic reviews) among primary study characteristics. However, only 1 of 30 reviews included age in a subgroup analysis, and 3 of 30 reviews identified the effects of sex, gender, or age as evidence gaps.

**Conclusions:**

This overview shows that the effects of sex, gender, or age were rarely considered in 30 systematic reviews of digital technologies for COPD treatment and monitoring. Furthermore, systematic reviews did not incorporate sex, gender, nor age in their implications for clinical practice. We recommend that future systematic reviews should (1) evaluate the effects of sex, gender, or age on the outcomes of digital technologies for treatment and monitoring of COPD and (2) better adhere to reporting guidelines to improve the confidence in review results.

**Trial Registration:**

PROSPERO CRD42022322924; https://www.crd.york.ac.uk/prospero/display_record.php?RecordID=322924

**International Registered Report Identifier (IRRID):**

RR2-10.2196/40538

## Introduction

Chronic obstructive pulmonary disease (COPD) is a chronic disease with an estimated global prevalence of 10.3% (391.9 million) among people aged 30 years to 79 years in 2019 [1]. COPD is a heterogeneous lung condition characterized by chronic respiratory symptoms due to abnormalities of the airways or alveoli (emphysema) that cause persistent, often progressive, airflow obstruction [2]. Especially in more advanced stages, there is a significant negative impact on quality of life, and the disease is associated with premature death [3,4]. COPD is also associated with a high economic burden that was estimated at €38.6 billion in the European Union alone [2]. Based on dynamic modeling, it has been predicted that women incur higher direct costs and lose more quality-adjusted life years than men [5]. The greatest proportion of the total costs in the health care system is accounted for by COPD exacerbations [2].

Sociodemographic factors, such as biological sex and age, play an important role in various aspects of COPD, including prevalence, deaths, and disability-adjusted life years according to The Global Burden of Disease Study 2019 [6]. In COPD literature, sex (a biological construct) is interchangeably referred to as gender (a social construct), and it is difficult to separate both terms because the constructs are multidimensional and interrelated [7]. Specifically, some COPD studies refer exclusively to sex [8,9], others refer exclusively or predominantly to gender [10,11], yet others use both terms [12,13]. Thus, it is important to consider all 3 factors (sex or gender and age) in the context of COPD [14].

In general, as individuals age, the likelihood of developing the condition increases [1,15], and diagnostic and therapeutic approaches vary depending on sociodemographic factors (sex, gender, or age) [8-13,16-19]. For example, although women tend to exhibit more severe symptoms of COPD than men throughout their lifespan [13], they may also respond better to specific treatments [9]. In addition, being female is linked to the development of severe early-onset COPD [8]. Interestingly, despite the higher disease severity, older COPD patients (ie, 65 years or older) seem to have a better quality of life and report fewer exacerbations than younger patients (ie, younger than 65 years), according to 2 large cohort studies reported together in 1 publication [20].

There are various treatment and monitoring options for COPD, depending on the stage and concomitant diseases, including therapies aiming at smoking cessation, pharmacological therapy, rehabilitation, self-management, and integrated care programs [2]. Digital technologies can provide support for various treatment options and assist with monitoring of chronic diseases by targeting patient needs or health care providers [21-24]. In terms of COPD, digital technologies targeting patient needs could deliver health information, alerts, and reminders based on health status and demographics. One example is a mobile app for COPD patients aged 40 years to 80 years that focuses on self-management behaviors, quality of life, and sustained behavior change, including physical activity promotion and smoking cessation [25]. Digital technologies targeting health care providers can assist with remote consultations or remote monitoring of health status. Examples of such technologies include telemonitoring to reduce hospitalizations due to COPD exacerbations and to improve quality of life in COPD patients aged 60 years or older [26] or remote (home-based) pulmonary rehabilitation to improve walking capacity [27].

Several systematic reviews have already addressed digital technology use in the context of COPD (eg, [28,29]). In general, systematic reviews should comprehensively and objectively evaluate existing evidence by assessing any potential effects of sociodemographic factors, such as sex, gender, or age, on health care outcomes [30]. It is unclear if and how systematic reviews considered the sociodemographic factors in the context of digital technologies for treatment and monitoring of COPD. The consideration of sex, gender, or age diversity could yield several potential benefits in this field. First, it could show if these factors are included in the evaluation of the effectiveness and safety of digital technology use in COPD. Second, it could provide the evidence necessary to develop digital interventions that are tailored to the individual needs of patients. For example, digital technologies for COPD could be made more user-friendly, thereby increasing their acceptance and use adherence. Third, it addresses the issues of equality and inclusion in health care, aligning with the principles of diversity or individualized medicine.

This study is an overview (ie, a systematic review of systematic reviews). In general, an overview allows the assessment and mapping of the existing evidence to identify evidence gaps that could be addressed in future systematic reviews of primary studies and thus aims to reduce the production of redundant systematic reviews. Typical overviews assess and compare the outcomes of multiple systematic reviews with similar population types or interventions [31]. This overview focuses on systematic reviews with the same populations (ie, people with COPD) and similar interventions (ie, digital technologies for treatment or monitoring of COPD). However, unlike a typical overview, we do not focus on the outcomes of such interventions (ie, if they are effective for treatment or monitoring of COPD). Instead, this study aimed to assess if systematic reviews considered the effects of sex, gender, or age on the outcomes of digital technologies for treatment and monitoring of COPD through an overview of such systematic reviews. The objectives of this overview were to (1) describe the definitions of sex or gender used in reviews; (2) determine whether the consideration of sex, gender, or age was planned in reviews; (3) determine whether sex, gender, or age was reported in review results; (4) determine whether sex, gender, or age was incorporated in implications for clinical practice in reviews; and (5) create an evidence map for the development of individualized clinical recommendations for COPD based on sex, gender, or age diversity.

## Methods

### Study Design

This study is an overview of systematic reviews and adheres to the PRIOR (Preferred Reporting Items for Overviews of Reviews) statement [32]. The PRIOR checklist is reported in Table S1 in Multimedia Appendix 1.

### Protocol and Registration

The protocol for this overview was prospectively registered in PROSPERO and published [14]. We adhered to the protocol except for 1 deviation. We planned to include systematic reviews if the confidence in their results was rated as moderate to high based on appraisals with AMSTAR 2 (A Measurement Tool to Assess Systematic Reviews, version 2) [33]. This inclusion criterion was omitted in this overview because too few systematic reviews received such ratings.

### Patient and Public Involvement

Patients and the public were not involved in the design nor conduct of this study. Therefore, no ethical approval was required for this overview.

### Eligibility Criteria

The eligibility criteria were reported in detail in our protocol [14]. The inclusion criteria for this overview were based on the PICOS (population, intervention, comparison, outcome, study type) framework: population (COPD), intervention (any intervention for treatment or monitoring of COPD supported by digital technologies), comparison (any other intervention or no intervention), outcome (any), and study (systematic review with reproducible methodology published in a peer-reviewed journal in English or German).

### Information Sources

The information sources in this overview were (1) international databases (MEDLINE via PubMed, Cochrane Library, Epistemonikos, and Web of Science) and (2) the reference sections of systematic reviews included in our overview.

### Search Strategy

The electronic search strategy (Table S2 in Multimedia Appendix 1) was developed, and the search was performed under the supervision of an experienced librarian. The electronic search was performed by the first author from database inception to June 1, 2022, without any limits. Bibliographic searches of the reference sections of included systematic reviews were performed by 2 authors, and final consensus was reached by discussion.

### Study Selection Process

Records identified in electronic and bibliographic searches were stored and processed in EndNote 20 (Clarivate). Studies were selected independently by 2 authors based on title and abstract screening, and full-text screening was performed in Covidence (Veritas Health Innovation). Consensus was reached by discussion. The list of excluded studies after full-text inspection with exclusion reasons is shown in Table S3 in Multimedia Appendix 1.

### Data Collection Process

Data were collected (ie, extracted from the included systematic reviews) into a self-developed spreadsheet (Excel, version 10; Microsoft Inc) that was pilot-tested and calibrated within the team. For this purpose, the spreadsheet was first created by 1 author, the data from 1 systematic review were extracted, and all authors provided feedback on whether the data items were complete and the extracted data were comprehensible and unambiguous. Subsequently, data from 5 systematic reviews were extracted by 2 authors independently. Once consensus was reached by discussion, the data from all systematic reviews were extracted by 1 author and checked by another author.

### Data Items

Data items included systematic review characteristics; PICOS criteria; and variables related to sex, gender, or age (Textbox 1).

**Data items in this overview of systematic reviews.**

**Data items:**

- Bibliographic information (eg, first author name, publication year)
- Population characteristics (eg, chronic obstructive pulmonary disease [COPD] diagnosis, definition of sex or gender)
- Intervention details (eg, digital technology type or device, such as a mobile app)
- Comparison type (eg, care as usual)
- Outcome type (eg, hospitalizations due to COPD exacerbations, quality of life)
- Systematic review type: Cochrane or non-Cochrane review
- Systematic review aim according to review authors
- Primary studies in the systematic review (number of studies, designs, and overlap among published studies)
- Risk of bias in primary studies according to review authors
- Data items for sex, gender, or age (eg, planned or performed subgroup or sensitivity analyses of outcomes based on sex, gender, or age)

### Risk of Bias Assessment (Critical Appraisal of Systematic Reviews)

We performed critical appraisals of systematic reviews using AMSTAR 2 [33] based on methods explained in the protocol [14]. AMSTAR 2 consists of 16 items that assess if various aspects of systematic reviews were fulfilled (ie, appropriately reported), including the steps involved in review preparation, literature search and study selection, and data extraction and analysis, as well as the information on any risks (eg, the risk of bias, publication bias, or sources of funding) [33]. The outcome of the critical appraisal is a confidence rating in the results of the systematic review (high, moderate, low, or critically low) that is assigned based on the type and the number of weaknesses (ie, not fulfilled items) in a review [33].

Two authors appraised all systematic reviews independently in Covidence and reached consensus by discussion.

### Overlap in Primary Studies Included in Systematic Reviews

Systematic reviews on the same topic could include the same primary studies. Such potential overlap was assessed by the creation of a citation matrix and the calculation of the overall corrected covered area (CCA) using the GROOVE (Graphical Representation of Overlap for Overviews) tool [34]. The GROOVE tool uses the calculation method introduced by Pieper et al [35] and their suggestion for interpretation, in which a CCA of 0% to 5% represents a slight overlap, 6% to 10% a moderate overlap, 11% to 15% a high overlap, and higher than 15% a very high overlap in primary studies cited in multiple systematic reviews.

### Data Synthesis Methods

The extracted data were synthesized using descriptive statistics (absolute and relative frequencies) or narratively by identifying common themes. For example, each review aim was read by 1 author to identify any information on sex, gender, or age. This information was subsequently quantified to cluster the reviews into groups (eg, age included in the review aim: yes or no). Another author checked the clustering.

We planned to perform subgroup analyses to assess if considerations of sex, gender, or age in systematic reviews are associated with systematic review type or appraisal rating on AMSTAR 2 [14]. These analyses aimed to compare the proportions of systematic reviews that considered sex, gender, or age (yes or no) with (1) systematic review type (Cochrane vs non-Cochrane) and (2) AMSTAR 2 confidence rating (high vs moderate) using chi-square tests and odds ratios with 95% confidence intervals.

### Reporting Bias or Certainty Assessment

The outcomes of the risk of bias assessment (the overall confidence ratings on AMSTAR 2) were reported for each systematic review and summarized using relative frequencies for all systematic reviews.

## Results

### Included Studies

#### Study Selection

**Figure 1:**
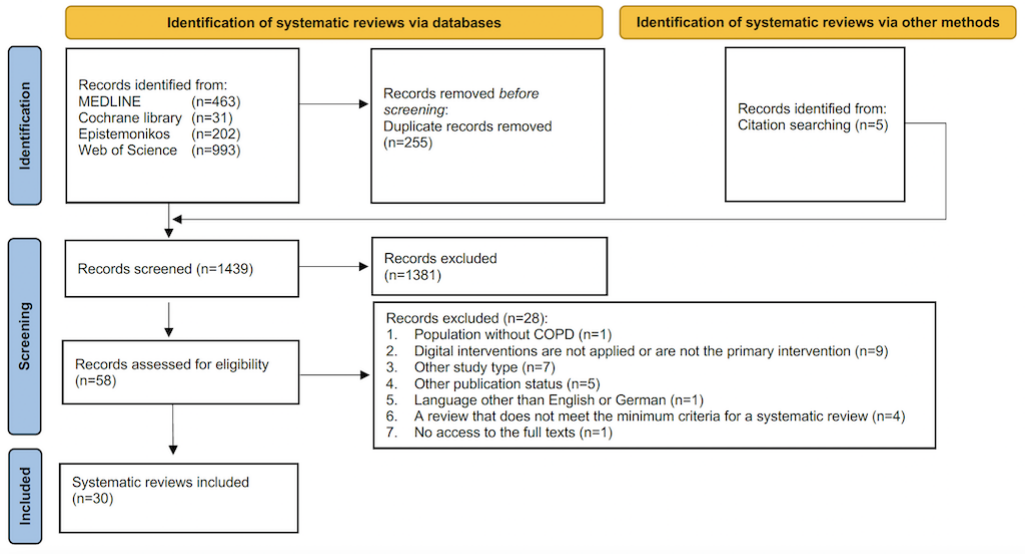
Flow diagram of study selection based on PRIOR (Preferred Reporting Items for Overviews of Reviews) guideline. COPD: chronic obstructive pulmonary disease.

Overall, from 1439 records (1434 from electronic searches and 5 from bibliographic searches), 30 systematic reviews [36-65] were included in this overview (Figure 1).

#### Characteristics of Systematic Reviews

All data are reported in Multimedia Appendix 2, and the detailed characteristics of the individual systematic reviews are shown in Table S4 in Multimedia Appendix 1. The synthesis of study characteristics in all 30 systematic reviews is shown in Table 1. The included 30 systematic reviews were published from 2010 to 2022, and most originated from Europe (13/30, 43%) or Asia (10/30, 33%). The number of primary studies included in the systematic reviews ranged from 3 to 38. All systematic reviews addressed any digital technologies for treatment or monitoring of COPD (eg, telemonitoring, telerehabilitation, mobile phone apps for self-management). Patients with COPD were included in all systematic reviews. Most systematic reviews (19/30, 63%) did not report the criteria for COPD diagnosis, while others used established diagnostic criteria for COPD [2] (8/30, 27%) or clinical diagnosis (3/30, 10%). Most systematic reviews (21/30, 70%) specified at least one primary outcome, including exacerbation rates, hospital admissions, exercise capacity, quality of life, patients’ adherence, or satisfaction. Digital technologies were compared with different control conditions, including care as usual or any kind of comparator, such as nondigital interventions. Most systematic reviews (16/30, 53%) included primary studies with any design, including randomized controlled trials (RCTS) and non-RCTs, and 17% (5/30) were Cochrane reviews. Most systematic reviews (27/30, 90%) appraised the risk of bias in primary studies, mainly with the Cochrane Risk of Bias tool version 1 or 2 (18/27, 67%). The systematic reviews evaluated digital technologies for COPD focusing on user outcomes, including efficacy or effectiveness (25/30, 83%) and acceptance, satisfaction, or adherence (3/30, 10%) or other outcomes (ie, evaluation of telemedicine focusing on providers and technology or a narrative synthesis of studies with telehealth technology).

**Table 1:** Characteristics of systematic reviews (n=30).

Characteristics		Results, n (%)
**Population**		
	COPD^a^ in all or majority of primary study participants	30 (100)
**Intervention (any digital intervention)**		
	Telehealth or telemedicine	10 (33)
	Telemonitoring	7 (23)
	Telehealth pulmonary rehabilitation	3 (10)
	Mobile device app interventions	4 (13)
	Digital interventions for self-management	2 (7)
	Other (eg, telenursing, remote respiratory assessments)	4 (13)
**Comparison**		
	Usual care	12 (40)
	Not specified	10 (33)
	Any comparator	3 (10)
	Other (eg, non-web-based interventions, center-based outpatient or inpatient pulmonary rehabilitation)	5 (17)
**Outcomes (primary outcome specified)^b^**		
	Resource use (eg, hospital admissions and readmissions, emergency department presentations)	11 (52)
	Quality of life (generic or disease-specific)	9 (43)
	Exacerbations	7 (33)
	Dyspnea	5 (24)
	Physical activity or exercise capacity	5 (24)
	Mortality	2 (10)
	Treatment adherence	2 (10)
	Other (eg, satisfaction, acceptance, adverse events)	8 (38)
**Study type**		
	Cochrane Review	5 (17)
	Other	25 (83)
**Study design of the included primary studies**		
	Only RCTs^c^	14 (47)
	RCTs/NRSI^d^	16 (53)
**Appraisal of the risk of bias in the primary studies**		
	Yes	27 (90)
	No information	3 (10)
**Appraisal instrument for risk of bias in primary studies^e^**		
	Cochrane Risk of Bias Tool 1 or 2	18 (67)
	Modified version of a scoring system to evaluate telemedicine research	4 (15)
	Own criteria	3 (11)
	Other (USPSTF^f^ Quality Rating Criteria, the Evidence Project risk of bias tool)	2 (7)

^a^COPD: chronic obstructive pulmonary disease.

^b^n=21.

^c^RCTs: randomized controlled trials.

^d^NRSI: nonrandomized studies of interventions.

^e^n=27.

^f^USPSTF: United States Preventive Services Task Force.

### Consideration of Sex, Gender, or Age in Systematic Reviews

#### Objective 1: Terminology and Definitions of Sex or Gender Used in Reviews

The terms sex and gender were not defined in any systematic review (Table 2).

#### Objective 2: Consideration of Sex, Gender, or Age Planned in Reviews

The influence of sex or gender was not considered in the aims nor planned analyses in any systematic review (Table 2). Of the 30 systematic reviews, age was included in the aim of 1 (3%) [36]. The purpose of this review was to examine factors (including age) that might influence overall acceptance of and dropout rates from telehealth interventions (eg, telemonitoring, telerehabilitation) [36]. Age diversity was planned to be investigated in a subgroup analysis in 3 of the 30 (10%) systematic reviews [36,57,58].

**Table 2:** Sex, gender, or age considerations in systematic reviews (n=30).

Systematic review section			Yes, n (%)
**Background, introduction, aims, or objectives**			
	Term sex or gender used	0	
	Terminology and definitions for sex or gender given	0	
	Sex or gender included in the review aim	0	
	Age included in the review aim	1 (3)	
**Methods**			
	Separate (subgroup) analysis by sex or gender planned	0	
	Separate (subgroup) analysis by age planned	3 (10)	
**Results**			
	Separate data by sex or gender reported	0	
	Separate data by age groups reported	1 (3)	
	Does the systematic review note that planned subgroup analyses by sex or gender could not be done and, if so, provides reasons?	0	
	Does the systematic review note that planned subgroup analyses by age could not be done and, if so, provides reasons?	2 (7)	
	Does the systematic review report that the primary studies analyzed or failed to analyze results by sex or gender?	0	
	Does the systematic review report that the primary studies analyzed or failed to analyze results by age?	2 (7)	
	Does the systematic review report sex or gender of the samples in the primary studies (eg, among the characteristics of the included studies)?	19 (63)	
	Does the systematic review report age of the samples in the primary studies (eg, among the characteristics of the included studies)?	25 (83)	
	Subgroup analysis other than by sex, gender, or age reported	7 (23)	
**Discussion/conclusion**			
	Does the systematic review consider sex or gender in discussion and conclusion?	0	
	Does the systematic review consider age in discussion and conclusion?	2 (7)	
	Does the review include sex or gender in the implications for clinical practice?	0	
	Does the review include age in the implications for clinical practice?	0	
	Does the systematic review include sex or gender as part of evidence gaps or suggestions for future research?	1 (3)	
	Does the systematic review include age as part of evidence gaps or suggestions for future research?	3 (10)	

#### Objective 3: Consideration of Sex, Gender, or Age Reported in Review Results

Most systematic reviews reported sex or gender (19/30, 63%) or age (25/30, 83%) among the sample characteristics extracted from primary studies (Table 2). Despite these data, the influence of sex or gender was not considered in the results of any systematic review (Table 2). Subgroup analysis including age was planned in 3 of the 30 (10%) systematic reviews [36,57,58] but was performed in only 1 systematic review [36]. This systematic review found comparable acceptance and dropout rates of telehealth measures in COPD in different age groups (younger or older than 69 years) [36]. The other 2 systematic reviews [57,58] did not perform the planned analyses due to a lack of data in the primary studies. Some systematic reviews (7/30, 23%) planned or performed subgroup analyses using other sample characteristics than sex, gender, or age, such as COPD severity, ethnicity or socioeconomic status, cognitive function, different types or duration of interventions, or different follow-up periods on the outcomes of digital technologies [49-51,53,54,63,64].

#### Objective 4: Consideration of Sex, Gender, or Age in Implications for Clinical Practice in Reviews

Sex or gender was not considered in the discussion and conclusion of results in any systematic review (Table 2). One systematic review [64] suggested that a stratified analysis should be conducted according to patient characteristics, including sex, in future research (Table 2). The same review [64] and 2 other systematic reviews [38,57] mentioned age as part of evidence gaps or suggestions for future research.

#### Objective 5: Evidence Map for Development of Individualized Clinical Recommendations for COPD Based on Sex, Gender, or Age Diversity

Based on inadequate evidence from systematic reviews, an evidence map with individualized recommendations for digital technology use in COPD based on sex, gender, or age could not be developed in this overview.

### Critical Appraisal of Individual Sources of Evidence

The critical appraisals based on AMSTAR 2 showed that the overall confidence in the results of the systematic reviews was high in 10% (3/30) of systematic reviews, moderate in 3.3% (1/30) of systematic reviews, low in 3.3% (1/30) of systematic reviews, and critically low in 83% (25/30) of systematic reviews (Table 3).

**Table 3:** Critical appraisal outcomes based on the AMSTAR 2 (A Measurement Tool to Assess Systematic Reviews) tool.

Number	Systematic review (citation)	Overall confidence in the results of the review
1	Alghamdi et al (2021) [36]	Critically low
2	Almojaibel (2016) [37]	Critically low
3	Alwashmi et al (2016) [38]	Critically low
4	Baroi et al (2018) [39]	Critically low
5	Bolton et al (2011) [40]	Critically low
6	Bonnevie et al (2021) [41]	Critically low
7	Calvache-Mateo et al (2021) [42]	Critically low
8	Cox et al (2021) [43]	High
9	Cruz et al (2014) [44]	Critically low
10	Cruz et al (2014) [45]	Critically low
11	Gregersen et al (2016) [46]	Critically low
12	Hong and Lee (2019) [47]	Critically low
13	Jang et al (2021) [48]	Critically low
14	Janjua et al (2021) [49]	High
15	Janjua et al (2021) [50]	High
16	Kamei et al (2013) [51]	Critically low
17	Kruse et al (2019) [52]	Critically low
18	Liu et al (2020) [53]	Critically low
19	Lu et al (2021) [54]	Critically low
20	Lundell et al (2015) [55]	Critically low
21	Martínez-García et al (2017) [56]	Critically low
22	McCabe et al (2017) [57]	Moderate
23	McLean et al (2011) [58]	Low
24	Michaelchuk et al (2022) [59]	Critically low
25	Polisena et al (2010) [60]	Critically low
26	Sabahi et al (2021) [61]	Critically low
27	Shaw et al (2020) [62]	Critically low
28	Song et al (2022) [63]	Critically low
29	Sul et al (2020) [64]	Critically low
30	Yang et al (2018) [65]	Critically low

According to the individual item ratings on AMSTAR 2 (Multimedia Appendix 3), the 3 most common weaknesses in the 30 systematic reviews were that the sources of funding for the primary studies included in the review were not reported (26/30, 87%), a list of excluded studies was absent (25/30, 83%), and a review protocol was not mentioned (18/30, 60%).

### Overlap in Primary Studies Included in Systematic Reviews

**Figure 2:**
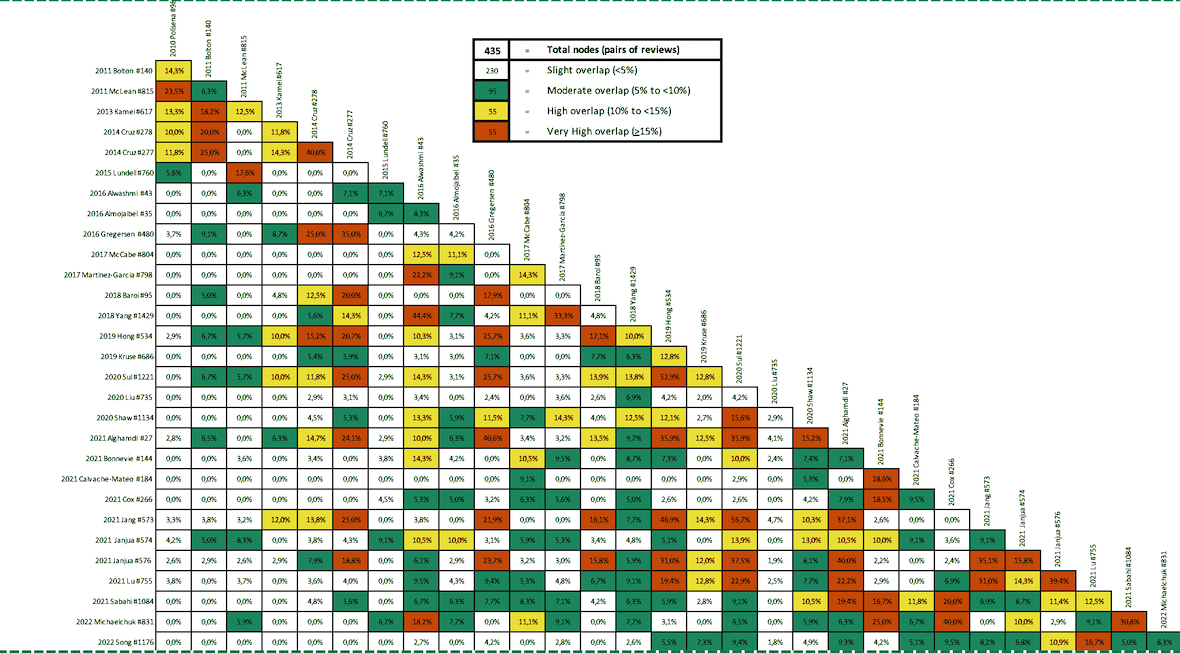
Overlap in the primary studies among review pairs based on the GROOVE (Graphical Representation of Overlap for Overviews) tool.

The overlap assessment showed that most primary studies were cited only once in any systematic review. Overall, there was a slight (ie, low) overlap in 182 primary studies included in the 30 systematic reviews (CCA of 4.21). The comparison between any 2 systematic reviews showed that the overlap was low (<5%) in 281 of 435 (64.6%) review pairs, moderate (5% to <10%) in 65 of 435 (14.9%) review pairs, high (10% to <15%) in 47 of 435 (10.8%) review pairs, and very high (≥15%) in 42 of 435 (9.6%) review pairs (Figure 2 and Multimedia Appendix 4).

### Subgroup Analyses

Subgroup analyses were planned in the protocol [14] but could not be performed due to the lack of sufficient data. Specifically, in this overview, the numbers of systematic reviews with high (3/30) or moderate (1/30) confidence ratings and considerations of sex (0/30), gender (0/30), or age (3/30) were too small to compute the odds ratios (ie, at least 1 cell in the contingency table was zero).

## Discussion

### Principal Findings

This overview included 30 systematic reviews that addressed any digital technologies for treatment and monitoring of COPD (eg, telemonitoring, telerehabilitation, or mobile phone apps for self-management). Although we did not limit the publication date in our search, the oldest included systematic review was published in 2010, and the newest was published in 2022. Although the confidence in the results of most reviews (25/30, 83%) was critically low, most reviews included different primary studies (ie, the overlap among primary studies included in the reviews was low).

Although data on sex, gender, or age were extracted from primary studies and reported in most systematic reviews among the primary study characteristics, these variables were rarely considered either in data analysis or explanation of results. The only systematic review with relevant results compared acceptance and dropout rates and found no differences between different age groups (younger or older than 69 years) [36] but had a critically low overall confidence rating according to AMSTAR 2 [33]. Two other systematic reviews planned subgroup analyses with different age groups but were unable to conduct them because of inadequate data [57,58]. None of the systematic reviews considered the effects of sex or gender in the objectives or planned analyses. This is surprising, since most reviews focused on user outcomes that potentially depend on sex, gender, or age, such as efficacy or effectiveness, acceptance, satisfaction, and adherence to digital technologies for COPD. Furthermore, systematic reviews did not incorporate sex, gender, nor age in their implications for clinical practice or policy and regulatory development.

### Comparison With Prior Work

Our results are consistent with previous findings. Although there is increasing acknowledgment that sex, gender, or age should be considered when designing and reporting research [66-69], multiple methodological studies suggest that there is still room for improvement [70-74]. For example, a cross-disciplinary bibliometric analysis found that, although more sex or gender-related information has been published in clinical research and public health over the past 4 decades, sex or gender continues to be underreported in biomedical studies [75]. A recent methodological study evaluating a sample 517 Cochrane reviews of interventions found that overall sex or gender consideration in Cochrane reviews was inadequate [74] because only 2.7% of Cochrane reviews reported sex in all review sections (ie, abstract, methods, descriptive results, analytical results, and discussion).

The limited data available on sex or gender in primary studies [76,77] may explain why these variables are also not reported in systematic reviews. Nevertheless, review authors could at least discuss such limitations, as was done in only 1 systematic review in our analysis [64]. Sex, gender, and age are important in COPD because these factors are associated with COPD prevalence, deaths, and disability-adjusted life years according to The Global Burden of Disease Study 2019 [6]. Furthermore, the use of digital technologies in the context of COPD may also depend on these factors. For example, 2 primary studies reported that dropouts from digital interventions for COPD were more likely to be female and older [78,79].

In general, sample diversity should be considered in systematic reviews of health care interventions. Factors including sex, gender, age, and other characteristics that encompass place of residence, race, ethnicity, culture, language, occupation, religion, education, socioeconomic status, social capital, and other factors such as sexual orientation or disability may all contribute to the experience of health inequity (Cochrane Handbook, Version 6.3, Chapter 16: Equity and specific populations [30]). Furthermore, although not addressed in this overview, eHealth literacy should also be addressed as part of sample diversity because digital health technology use depends on eHealth literacy [80]. Therefore, it is necessary that these factors are also adequately considered in the preparation of systematic reviews. Future systematic reviews should prioritize adherence to international reporting guidelines concerning sex and gender equity, as advocated by Heidari et al [81], or using the extension of the PRISMA (Preferred Reporting Items for Systematic Reviews and Meta-Analyses) statement for equity-focused systematic reviews (PRISMA-E 2012) [82]. To address health equity in systematic reviews, review authors, editors, and funding organizations should demand more rigorous analysis and reporting related to sample diversity, at least in terms of basic sample characteristics including sex, gender, or age.

### Evidence Appraisal

We performed an appraisal of the 30 systematic reviews included in our overview with the AMSTAR 2 tool. Our analysis revealed that there is room for improvement in the overall methodological quality of systematic reviews on digital technologies used for treatment or monitoring of COPD. As we have already suggested, better adherence to established reporting guidelines for systematic reviews and prospective registration of review protocols could increase overall confidence in the results of systematic reviews on digital technologies for the treatment and monitoring of COPD [83]. As financial interests may exist in the field of digital interventions, systematic reviews should document the sources of funding for primary studies [80]. Our findings are consistent with those of other studies that evaluated the methodological quality of systematic reviews in various areas related to digital interventions [84-86] and showed that the majority or all of the included systematic reviews on digital interventions had low methodological quality, resulting in critically low overall confidence in their results. Consequently, such systematic reviews may have little practical use for clinical decisions or policy development according to AMSTAR 2 [33].

### Strengths and Limitations

This is the first overview that assessed if sex, gender, or age is considered when evaluating the outcomes of digital technologies for the treatment and monitoring of COPD. We followed our published protocol [14] and used a comprehensive search strategy to identify relevant systematic reviews. Additionally, all critical steps were performed independently by at least 2 researchers, thereby improving the consistency of our findings. The overview also has some limitations. The protocol for this study has undergone rigorous development, including iterative testing and revision of the electronic search syntax by an experienced database specialist. However, there is a possibility that some relevant systematic reviews in the new field of digital technologies were missed in the electronic search. To address this limitation, a manual search of bibliographies of the included systematic reviews was performed to find additional literature. Although the search was performed for systematic reviews in English or German, only 1 systematic review was excluded due to language in the full-text screen. Furthermore, we planned to include systematic reviews with moderate or high confidence ratings based on the appraisals of systematic reviews with the AMSTAR 2 tool [33]. Since most systematic reviews received low or critically low confidence ratings, we included all identified systematic reviews in this overview. Consequently, the overview includes systematic reviews with poor (low or critically low) confidence ratings. Finally, our overview focuses on the potential impact of only 3 variables (sex or gender and age) on outcomes of digital technologies in COPD. Nonetheless, it may be worthwhile to explore several other participant characteristics in COPD, including age at onset [8], race [87], as well as education and socioeconomic status [88].

### Conclusion

This overview shows that the effects of sex, gender, or age were rarely considered in 30 systematic reviews of digital technologies for the treatment and monitoring of COPD. Furthermore, systematic reviews did not incorporate sex, gender, nor age in their implications for clinical practice or policy and regulatory development. We recommend that future systematic reviews should (1) evaluate the effects of sex, gender, or age on the outcomes of digital technologies for the treatment and monitoring of COPD and (2) better adhere to reporting guidelines to improve the confidence in review results.

## Appendix

Multimedia Appendix 1 PRIOR checklist and supplementary tables.

Multimedia Appendix 2 Data file.

Multimedia Appendix 3 Item ratings on AMSTAR 2 in 30 systematic reviews.

Multimedia Appendix 4 Overall results of overlap for overviews with the GROOVE tool.

## Abbreviations

AMSTAR 2: A Measurement Tool to Assess Systematic Reviews, version 2
CCA: corrected covered area
COPD: chronic obstructive pulmonary disease
GROOVE: Graphical Representation of Overlap for OVErviews
PICOS: population, intervention, comparison, outcome, study type
PRIOR: Preferred Reporting Items for Overviews of Reviews statement
PRISMA-E: equity extension of the Preferred Reporting Items for Systematic Reviews and Meta-Analyses
RCT: randomized controlled trial

## Data Availability

This overview of systematic reviews is based on previously published data. All relevant data are reported in the article and appendices.
