# Supplementary material for: Consideration of Sex, Gender, or Age on Outcomes of Digital Technologies for Treatment and Monitoring of Chronic Obstructive Pulmonary Disease: Overview of Systematic Reviews
Source: J Med Internet Res. 2023 Nov 29;25:e49639. doi: 10.2196/49639 (PMC10719824; doi:10.2196/49639)
Supplement: Multimedia Appendix 1 [file jmir_v25i1e49639_app1.docx]

Multimedia Appendix 1. Supplementary tables.

Contents

[Table S1. PRIOR Checklist. 2](#_Toc150613929)

[Table S2. Search strategy 5](#_Toc150613930)

[Table S3. Table of excluded studies. 7](#_Toc150613931)

[Table S4. Characteristics of the included systematic reviews. 9](#_Toc150613932)

## Table S1. PRIOR Checklist.

| **Section**  Topic | **#** | **Item** | **Location reported** |
| --- | --- | --- | --- |
| **TITLE** | | |  |
| Title | 1 | Identify the report as an overview of reviews. | Yes |
| **ABSTRACT** | | |  |
| Abstract | 2 | Provide a comprehensive and accurate summary of the purpose, methods, and results of the overview of reviews. | Yes |
| **INTRODUCTION** | | |  |
| Rationale | 3 | Describe the rationale for conducting the overview of reviews in the context of existing knowledge. | Yes |
| Objectives | 4 | Provide an explicit statement of the objective(s) or question(s) addressed by the overview of reviews. | Yes |
| **METHODS** | | |  |
| Eligibility criteria | 5a | Specify the inclusion and exclusion criteria for the overview of reviews. If supplemental primary studies were included, this should be stated, with a rationale. | Yes |
|  | 5b | Specify the definition of ‘systematic review’ as used in the inclusion criteria for the overview of reviews. | Yes |
| Information sources | 6 | Specify all databases, registers, websites, organizations, reference lists, and other sources searched or consulted to identify systematic reviews and supplemental primary studies (if included).  Specify the date when each source was last searched or consulted. | Yes |
| Search strategy | 7 | Present the full search strategies for all databases, registers and websites, such that they could be reproduced. Describe any search filters and limits applied. | Multimedia Appendix 1 |
| Selection process | 8a | Describe the methods used to decide whether a systematic review or supplemental primary study (if included) met the inclusion criteria of the overview of reviews. | Yes |
|  | 8b | Describe how overlap in the populations, interventions, comparators, and/or outcomes of systematic reviews was identified and managed during study selection. | NA |
| Data collection process | 9a | Describe the methods used to collect data from reports. | Yes |
|  | 9b | If applicable, describe the methods used to identify and manage primary study overlap at the level  of the comparison and outcome during data collection. For each outcome, specify the method used to illustrate and/or quantify the degree of primary study overlap across systematic reviews. | NA |
|  | 9c | If applicable, specify the methods used to manage discrepant data across systematic reviews during data collection. | NA |
| Data items | 10 | List and define all variables and outcomes for which data were sought. Describe any assumptions made and/or measures taken to identify and clarify missing or unclear information. | Yes |
| Risk of bias assessment | 11a | Describe the methods used to *assess* risk of bias or methodological quality of the included systematic reviews. | Yes |
|  | 11b | Describe the methods used to *collect* data on (from the systematic reviews) and/or *assess* the risk of bias of the primary studies included in the systematic reviews. Provide a justification for instances where flawed, incomplete, or missing assessments are identified but not re-assessed. | Yes |
|  | 11c | Describe the methods used to *assess* the risk of bias of supplemental primary studies (if included). | NA |
| Synthesis methods | 12a | Describe the methods used to summarize or synthesize results and provide a rationale for the choice(s). | NA |
|  | 12b | Describe any methods used to explore possible causes of heterogeneity among results. | NA |
|  | 12c | Describe any sensitivity analyses conducted to assess the robustness of the synthesized results. | NA |
| Reporting bias assessment | 13 | Describe the methods used to *collect* data on (from the systematic reviews) and/or *assess* the risk of bias due to missing results in a summary or synthesis (arising from reporting biases at the levels of the systematic reviews, primary studies, and supplemental primary studies, if included). | NA |
| Certainty assessment | 14 | Describe the methods used to *collect* data on (from the systematic reviews) and/or *assess* certainty (or confidence) in the body of evidence for an outcome. | NA |
| **RESULTS** | | |  |
| Systematic review and supplemental primary study selection | 15a | Describe the results of the search and selection process, including the number of records screened, assessed for eligibility, and included in the overview of reviews, ideally with a flow diagram. | Yes |
|  | 15b | Provide a list of studies that might appear to meet the inclusion criteria, but were excluded, with the main reason for exclusion. | Multimedia Appendix 1 |
| Characteristics of systematic reviews and  supplemental primary studies | 16 | Cite each included systematic review and supplemental primary study (if included) and present its characteristics. | Yes |
| Primary study overlap | 17 | Describe the extent of primary study overlap across the included systematic reviews. | Yes |
| Risk of bias in systematic reviews, primary studies, and  supplemental primary studies | 18a | Present assessments of risk of bias or methodological quality for each included systematic review. | Yes |
|  | 18b | Present assessments (*collected* from systematic reviews or *assessed* anew) of the risk of bias of  the primary studies included in the systematic reviews. | NA |
|  | 18c | Present assessments of the risk of bias of supplemental primary studies (if included). | NA |
| Summary or synthesis of results | 19a | For all outcomes, summarize the evidence from the systematic reviews and supplemental primary studies (if included). If meta-analyses were done, present for each the summary estimate and its  precision and measures of statistical heterogeneity. If comparing groups, describe the direction of the effect. | Yes |
|  | 19b | If meta-analyses were done, present results of all investigations of possible causes of  heterogeneity. | NA |
|  | 19c | If meta-analyses were done, present results of all sensitivity analyses conducted to assess the  robustness of synthesized results. | NA |
| Reporting biases | 20 | Present assessments (*collected* from systematic reviews and/or *assessed* anew) of the risk of bias due to missing primary studies, analyses, or results in a summary or synthesis (arising from reporting biases at the levels of the systematic reviews, primary studies, and supplemental primary  studies, if included) for each summary or synthesis assessed. | NA |
| Certainty of  evidence | 21 | Present assessments (*collected* or *assessed* anew) of certainty (or confidence) in the body of  evidence for each outcome. | NA |
| **DISCUSSION** | | |  |
| Discussion | 22a | Summarize the main findings, including any discrepancies in findings across the included systematic reviews and supplemental primary studies (if included). | Yes |
|  | 22b | Provide a general interpretation of the results in the context of other evidence. | Yes |
|  | 22c | Discuss any limitations of the evidence from systematic reviews, their primary studies, and supplemental primary studies (if included) included in the overview of reviews. Discuss any  limitations of the overview of reviews methods used. | Yes |
|  | 22d | Discuss implications for practice, policy, and future research (both systematic reviews and  primary research). Consider the relevance of the findings to the end users of the overview of reviews, e.g., healthcare providers, policymakers, patients, among others. | Yes |
| **OTHER INFORMATION** | | |  |
| Registration and protocol | 23a | Provide registration information for the overview of reviews, including register name and registration number, or state that the overview of reviews was not registered. | Yes |
|  | 23b | Indicate where the overview of reviews protocol can be accessed, or state that a protocol was not prepared. | Yes |
|  | 23c | Describe and explain any amendments to information provided at registration or in the protocol.  Indicate the stage of the overview of reviews at which amendments were made. | Yes |
| Support | 24 | Describe sources of financial or non-financial support for the overview of reviews, and the role of  the funders or sponsors in the overview of reviews. | Yes |
| Competing  interests | 25 | Declare any competing interests of the overview of reviews' authors. | Yes |
| Author information | 26a | Provide contact information for the corresponding author. | Yes |
|  | 26b | Describe the contributions of individual authors and identify the guarantor of the overview of reviews. | Yes |
| Availability of data and other materials | 27 | Report which of the following are available, where they can be found, and under which conditions they may be accessed: template data collection forms, data collected from included systematic reviews and supplemental primary studies; analytic code; any other materials used in the overview of reviews. | Multimedia Appendix 1-3 |

‘Yes’ means that the item was addressed in the subheading corresponding to section name in the checklist. NA=not applicable. Source: Gates M, Gates A, Pieper D, et al. Reporting guideline for overviews of reviews of healthcare interventions: development of the PRIOR statement. BMJ 2022;378:e070849. doi:10.1136/bmj-2022-070849.

## Table S2. Search strategy

| MEDLINE via PubMed, search date 1.6.2022 |
| --- |
| #1 ("pulmonary disease, chronic obstructive"[MeSH Terms] OR "chronic obstructive pulmonary disease*"[Title/Abstract] OR "chronic obstructive airways disease*"[Title/Abstract] OR "chronic obstructive lung disease*"[Title/Abstract] OR "COPD"[Title/Abstract] OR "COAD"[Title/Abstract])  #2 ("internet"[MeSH Terms] OR "telemedicine"[MeSH Terms] OR "mobile applications"[MeSH Terms] OR "medical informatics applications"[MeSH Terms] OR "Wearable Electronic Devices"[MeSH Terms] OR "computers, handheld"[MeSH Terms] OR ("digital*"[Title/Abstract] OR "internet*"[Title/Abstract] OR "web based"[Title/Abstract] OR "webbased"[Title/Abstract] OR "online"[Title/Abstract] OR "on-line"[Title/Abstract] OR "www"[Title/Abstract] OR "world wide web"[Title/Abstract] OR "website"[Title/Abstract] OR "web site"[Title/Abstract] OR "mobile"[Title/Abstract] OR "web-assisted"[Title/Abstract] OR ("telehealth*"[Title/Abstract] OR "telemed*"[Title/Abstract] OR "tele health*"[Title/Abstract] OR "tele med*"[Title/Abstract] OR "e health*"[Title/Abstract] OR "ehealth*"[Title/Abstract] OR "electronic health*"[Title/Abstract] OR "mhealth*"[Title/Abstract] OR "m health*"[Title/Abstract]) OR ("app"[Title/Abstract] OR "apps"[Title/Abstract] OR "medical application*"[Title/Abstract] OR "health application*"[Title/Abstract] OR "healthcare application*"[Title/Abstract] OR "phone application*"[Title/Abstract] OR "electronic application*"[Title/Abstract] OR "software application*"[Title/Abstract]) OR ("wearable*"[Title/Abstract] OR "wearability"[Title/Abstract]) OR ("tablet*"[Title/Abstract] OR (("handheld*"[Title/Abstract] OR "hand held*"[Title/Abstract]) AND "computer*"[Title/Abstract]) OR "smart phone*"[Title/Abstract] OR "smartphone*"[Title/Abstract] OR "cellphone*"[Title/Abstract] OR "cell phone*"[Title/Abstract] OR "smart device*"[Title/Abstract] OR "smart watch"[Title/Abstract] OR "apple watch"[Title/Abstract] OR "Iphone"[Title/Abstract] OR "Ipad"[Title/Abstract])))  #3 "systematic review"[Filter]  #1 AND #2 AND #3 |
| **Cochrane,** search date 1.6.2022 |
| ([mh "pulmonary disease, chronic obstructive"] OR ("chronic obstructive pulmonary disease":ti,ab OR "chronic obstructive airways disease":ti,ab OR "chronic obstructive lung disease":ti,ab OR COPD:ti,ab) OR COAD:ti,ab) AND ([mh internet] OR [mh telemedicine] OR [mh "mobile applications"] OR [mh "medical informatics applications"] OR [mh "Wearable Electronic Devices"] OR [mh "computers, handheld"] OR (digital*:ti,ab OR internet*:ti,ab OR "web based":ti,ab OR webbased:ti,ab OR online:ti,ab OR on-line:ti,ab OR www:ti,ab OR "world wide web":ti,ab OR website:ti,ab OR "web site":ti,ab OR mobile:ti,ab OR web-assisted:ti,ab OR (telehealth*:ti,ab OR telemed*:ti,ab OR ("tele" NEXT health*):ti,ab OR ("tele" NEXT med*):ti,ab OR ("e" NEXT health*):ti,ab OR ehealth*:ti,ab OR ("electronic" NEXT health*):ti,ab OR mhealth*:ti,ab OR ("m" NEXT health*):ti,ab) OR (app:ti,ab OR apps:ti,ab OR ("medical" NEXT application*):ti,ab OR ("health" NEXT application*):ti,ab OR ("healthcare" NEXT application*):ti,ab OR ("phone" NEXT application*):ti,ab OR ("electronic" NEXT application*):ti,ab OR ("software" NEXT application*):ti,ab) OR (wearable*:ti,ab OR wearability:ti,ab) OR (tablet*:ti,ab OR ((handheld*:ti,ab OR ("hand" NEXT held*):ti,ab) AND computer*:ti,ab) OR ("smart" NEXT phone*):ti,ab OR smartphone*:ti,ab OR cellphone*:ti,ab OR ("cell" NEXT phone*):ti,ab OR ("smart" NEXT device*):ti,ab OR "smart watch":ti,ab OR "apple watch":ti,ab OR Iphone:ti,ab OR Ipad:ti,ab))) |
| **Web of Science,** search date 1.6.2022 |
| #1 ALL=((pulmonary disease, chronic obstructive or "chronic obstructive pulmonary disease" or COPD or COAD or "chronic obstructive airways disease" or "chronic obstructive lung disease"))  #2 TI=((internet OR telemedicine OR "mobile applications" OR "medical informatics applications" OR "Wearable Electronic Devices" OR "computers, handheld" OR digital* OR internet* OR "web based" OR webbased OR online OR on-line OR www OR "world wide web" OR website OR "web site" OR mobile OR web-assisted OR telehealth* OR telemed* OR "tele health*" OR "tele med*" OR "e health*" OR ehealth* OR "electronic health*" OR mhealth* OR "m health*" OR app OR apps OR "medical application*" OR "health application*" OR "healthcare application*" OR "phone application*" OR "electronic application*" OR "software application*" OR wearable* OR wearability OR tablet* OR handheld* OR "hand held*" OR "smart phone*" OR smartphone* OR cellphone* OR "cell phone*" OR "smart device*" OR "smart watch" OR "apple watch" OR Iphone OR Ipad))  #1 AND #2 |
| **Epistemonikos,** search date 1.6.2022 |
| #1 (title:(chronic obstructive pulmonary disease) OR abstract:(chronic obstructive pulmonary disease)) OR (title:(COPD) OR abstract:(COPD)) OR (title:(pulmonary disease, chronic obstructive) OR abstract:(pulmonary disease, chronic obstructive)) OR (title:(chronic obstructive airways disease) OR abstract:(chronic obstructive airways disease)) OR (title:(COAD) OR abstract:(COAD)) OR (title:(chronic obstructive lung disease) OR abstract:(chronic obstructive lung disease))  #2 (title:(internet OR telemedicine OR "mobile applications" OR "medical informatics applications" OR "Wearable Electronic Devices" OR "computers, handheld" OR digital* OR internet* OR "web based" OR webbased OR online OR on-line OR www OR "world wide web" OR website OR "web site" OR mobile OR web-assisted OR telehealth* OR telemed* OR "tele health*" OR "tele med*" OR "e health*" OR ehealth* OR "electronic health*" OR mhealth* OR "m health*" OR app OR apps OR "medical application*" OR "health application*" OR "healthcare application*" OR "phone application*" OR "electronic application*" OR "software application*" OR wearable* OR wearability OR tablet* OR handheld* OR "hand held*" OR "smart phone*" OR smartphone* OR cellphone* OR "cell phone*" OR "smart device*" OR "smart watch" OR "apple watch" OR Iphone OR Ipad) OR abstract:(internet OR telemedicine OR "mobile applications" OR "medical informatics applications" OR "Wearable Electronic Devices" OR "computers, handheld" OR digital* OR internet* OR "web based" OR webbased OR online OR on-line OR www OR "world wide web" OR website OR "web site" OR mobile OR web-assisted OR telehealth* OR telemed* OR "tele health*" OR "tele med*" OR "e health*" OR ehealth* OR "electronic health*" OR mhealth* OR "m health*" OR app OR apps OR "medical application*" OR "health application*" OR "healthcare application*" OR "phone application*" OR "electronic application*" OR "software application*" OR wearable* OR wearability OR tablet* OR handheld* OR "hand held*" OR "smart phone*" OR smartphone* OR cellphone* OR "cell phone*" OR "smart device*" OR "smart watch" OR "apple watch" OR Iphone OR Ipad))  #1 AND #2 |

## Table S3. Table of excluded studies.

| **Study** | **Reason for exclusion** |
| --- | --- |
| Alghamdi SM, Alqahtani JS, Aldhahir AM, Alrajeh AM, Aldabayan YS. Effectiveness of Telehealth-Based Interventions with Chronic Obstructive Pulmonary Disease: A Systematic Review and Meta-Analysis. AMERICAN JOURNAL OF RESPIRATORY AND CRITICAL CARE MEDICINE. 2020;201. | 4 |
| Alrajeh A, Aldabayan Y, Pickett E, Quaderi S, Lipman M, Hurst J. Tele-Health with Chronic Obstructive Pulmonary Disease (COPD). AMERICAN JOURNAL OF RESPIRATORY AND CRITICAL CARE MEDICINE. 2018;197. | 7 |
| Barbosa MT, Sousa CS, Morais-Almeida M, Simoes MJ, Mendes P. Telemedicine in COPD: An Overview by Topics. COPD-JOURNAL OF CHRONIC OBSTRUCTIVE PULMONARY DISEASE. 2020;17(5):601-17. doi: 10.1080/15412555.2020.1815182. | 3 |
| Bartoli L, Zanaboni P, Masella C, Ursini N. Systematic review of telemedicine services for patients affected by chronic obstructive pulmonary disease (COPD). Telemed J E Health. 2009;15(9):877-83. doi: 10.1089/tmj.2009.0044. | 6 |
| Buekers J, De Boever P, Vaes AW, Aerts JM, Wouters EFM, Spruit MA, et al. Oxygen saturation measurements in telemonitoring of patients with COPD: a systematic review. Expert Rev Respir Med. 2018;12(2):113-23. doi: 10.1080/17476348.2018.1417842. | 2 |
| Burge AT, Cox NS, Abramson MJ, Holland AE. Interventions for promoting physical activity in people with chronic obstructive pulmonary disease (COPD). Cochrane Database Syst Rev. 2020;4(4):Cd012626. doi: 10.1002/14651858.CD012626.pub2. | 2 |
| Coll F, Cavalheri V, Gucciardi DF, Wulff S, Hill K. Quantifying the Effect of Monitor Wear Time and Monitor Type on the Estimate of Sedentary Time in People with COPD: Systematic Review and Meta-Analysis. J Clin Med. 2022;11(7). doi: 10.3390/jcm11071980. | 2 |
| Gaveikaite V, Grundstrom C, Winter S, Chouvarda I, Maglaveras N, Priori R. A systematic map and in-depth review of European telehealth interventions efficacy for chronic obstructive pulmonary disease. Respir Med. 2019;158:78-88. doi: 10.1016/j.rmed.2019.09.005. | 3 |
| Glynn L, Moloney E, Klooblall M, McCabe C, Mc Cann M. A review of the feasibility of a smartphone application (app) supporting a self-management programme among Chronic Obstructive Pulmonary Disease (COPD) patients. IRISH JOURNAL OF MEDICAL SCIENCE. 2021;190(SUPPL 1):S52-S3. | 4 |
| Gorst SL, Armitage CJ, Brownsell S, Hawley MS. Home telehealth uptake and continued use among heart failure and chronic obstructive pulmonary disease patients: a systematic review. Ann Behav Med. 2014;48(3):323-36. doi: 10.1007/s12160-014-9607-x. | 1 |
| Hallensleben C, van Luenen S, Rolink E, Ossebaard HC, Chavannes NH. eHealth for people with COPD in the Netherlands: a scoping review. International journal of chronic obstructive pulmonary disease. 2019;14:1681-90. doi: 10.2147/COPD.S207187. | 3 |
| Jakobsen AS, Laursen LC, Schou L, Emme C, Phanareth KV. [Varying effect of telemedicine in the treatment of chronic obstructive pulmonary disease--a systematic review]. Ugeskr Laeger. 2012;174(14):936-42. | 5 |
| Jiang WP, Wang LL, Song YL. Titration and follow-up for home noninvasive positive pressure ventilation in chronic obstructive pulmonary disease: The potential role of tele-monitoring and the Internet of things. CLINICAL RESPIRATORY JOURNAL. 2021;15(7):705-15. doi: 10.1111/crj.13352. | 2 |
| Konstantinidis A, Kyriakopoulos C, Ntritsos G, Giannakeas N, Gourgoulianis KI, Kostikas K, et al. The Role of Digital Tools in the Timely Diagnosis and Prevention of Acute Exacerbations of COPD: A Comprehensive Review of the Literature. DIAGNOSTICS. 2022;12(2). doi: 10.3390/diagnostics12020269. | 6 |
| Lao XD, Zhang J, Bai CX. The implication of telehealthcare in COPD management of China. EXPERT REVIEW OF RESPIRATORY MEDICINE. 2013;7(5):459-63. doi: 10.1586/17476348.2013.838019. | 3 |
| Lundell S, Holmner A, Rehn B, Nyberg A, Wadell K. Telehealthcare for patients with COPD, effects on physical activity level, physical capacity and dyspnea: A systematic review and meta-analysis. EUROPEAN RESPIRATORY JOURNAL. 2014;44. | 4 |
| Malaguti C, Dal Corso S, Janjua S, Holland AE. Supervised maintenance programmes following pulmonary rehabilitation compared to usual care for chronic obstructive pulmonary disease. Cochrane Database Syst Rev. 2021;8(8):Cd013569. doi: 10.1002/14651858.CD013569.pub2. | 2 |
| McLean S, Nurmatov U, Liu JL, Pagliari C, Car J, Sheikh A. Telehealthcare for chronic obstructive pulmonary disease: Cochrane Review and meta-analysis. Br J Gen Pract. 2012;62(604):e739-49. doi: 10.3399/bjgp12X658269. | 3 |
| Mehdipour A, Wiley E, Richardson J, Beauchamp M, Kuspinar A. The Performance of Digital Monitoring Devices for Oxygen Saturation and Respiratory Rate in COPD: A Systematic Review. Copd. 2021;18(4):469-75. doi: 10.1080/15412555.2021.1945021. | 2 |
| Murphy LA, Harrington P, Taylor SJ, Teljeur C, Smith SM, Pinnock H, et al. Clinical-effectiveness of self-management interventions in chronic obstructive pulmonary disease: An overview of reviews. Chron Respir Dis. 2017;14(3):276-88. doi: 10.1177/1479972316687208. | 3 |
| Parikh S, Kaye L, Gondalia R, Barrett M, Henderson K, Stempel D. SOCIAL CONNECTEDNESS AND ASSOCIATED DIGITAL HEALTH SOLUTIONS IN THE COPD PATIENT POPULATION: A SYSTEMATIC REVIEW. ANNALS OF BEHAVIORAL MEDICINE. 2019;53:S538-S. | 4 |
| Pedone C, Lelli D. Systematic review of telemonitoring in COPD: an update. Pneumonol Alergol Pol. 2015;83(6):476-84. doi: 10.5603/PiAP.2015.0077. | 6 |
| Pericleous P, van Staa TP. The use of wearable technology to monitor physical activity in patients with COPD: a literature review. International journal of chronic obstructive pulmonary disease. 2019;14:1317-22. doi: 10.2147/COPD.S193037. | 2 |
| Shaw G, Whelan M, Armitage L, Farmer A. Are mobile device applications effective at supporting COPD self-management compared to usual care? EUROPEAN RESPIRATORY JOURNAL. 2019;54. doi: 10.1183/13993003.congress-2019.PA747. | 4 |
| Smith SM, Holland AE, McDonald CF. Beyond forest plots: clinical gestalt and its influence on COPD telemonitoring studies and outcomes review. BMJ Open. 2019;9(12):e030779. doi: 10.1136/bmjopen-2019-030779. | 3 |
| Sobnath DD, Philip N, Kayyali R, Nabhani-Gebara S, Pierscionek B, Vaes AW, et al. Features of a Mobile Support App for Patients With Chronic Obstructive Pulmonary Disease: Literature Review and Current Applications. JMIR mHealth and uHealth. 2017;5(2):e17. doi: 10.2196/mhealth.4951. | 2 |
| Vitacca M, Montini A, Comini L. How will telemedicine change clinical practice in chronic obstructive pulmonary disease? THERAPEUTIC ADVANCES IN RESPIRATORY DISEASE. 2018;12. doi: 10.1177/1753465818754778. | 6 |
| Yang F, Xiong ZF, Yang C, Li L, Qiao G, Wang Y, et al. Continuity of Care to Prevent Readmissions for Patients with Chronic Obstructive Pulmonary Disease: A Systematic Review and Meta-Analysis. Copd. 2017;14(2):251-61. doi: 10.1080/15412555.2016.1256384. | 2 |

## Table S4. Characteristics of the included systematic reviews.

| Study ID /Region | Population | Intervention | Comparison | Primary outcome(s) | Study designs (Studies included) | Summary of age, sex or gender related findings |
| --- | --- | --- | --- | --- | --- | --- |
| Alghamdi 2021 [37] / Asia | COPD | telehealth intervention (e.g., telemonitoring, telerehabilitation) | not specified | (1) acceptance (2) dropout rates | RCTs^b^/NRSI^c^ (27) | comparable acceptance and dropout rates of telehealth measures in COPD below or above the age of 69 years, no consideration of sex or gender |
| Almojaibel 2016 [38] / North America | COPD | home-based real-time pulmonary rehabilitation services | not specified | not specified | RCTs/NRSI (7) | no consideration |
| Alwashmi 2016 [39] / North America | COPD | smartphone interventions | usual care | exacerbation | RCTs/NRSI (6) | age as part of suggestions for future research |
| Baroi 2018 [40] / Australia/New Zealand | COPD | remote respiratory assessments | not specified | not specified | RCTs/NRSI (15) | no consideration |
| Bolton 2021 [41] / Europe | COPD | telemonitoring | not specified | not specified | RCTs/NRSI (6) | no consideration |
| Bonnevie 2021 [42] / Europe | COPD | home-based exercise therapy (ET) delivered using advanced telehealth technology (ATT) | (1) no ET (2) in/outpatient ET (3) home-based ET without ATT | (1) exercise capacity (2) Quality of Life (general or disease-specific) (3) functional dyspnoea (4) Cost-effectiveness | RCTs (15) | no consideration |
| Calvache-Mateo 2021 [43] / Europe | COPD | web-based supportive interventions | non-web-based interventions | Quality of Life | RCTs (9) | no consideration |
| Cox 2021 [44] /  Australia/New Zealand | chronic respiratory disease (99% of people in studies had COPD) | telerehabilitation | (1) centre-based (outpatient) pulmonary rehabilitation (2) inpatient pulmonary rehabilitation (3) no rehabilitation control | (1) Exercise capacity (2) Adverse events (3) Dyspnoea (4) Quality of Life (generic or disease-specific) | RCTs/NRSI (15) | no consideration |
| Cruz 2014a [45] / Europe | COPD | telemonitoring | not specified | (1) patients’ adherence (2) satisfaction | RCTs/NRSI (12) | no consideration |
| Cruz 2014b [46] / Europe | COPD | telemonitoring | usual care | not specified | RCTs/NRSI (9) | no consideration |
| Gregersen 2016 [47] / Europe | COPD | telehealth intervention (e.g., telemonitoring, telerehabilitation) | not specified | Quality of Life | RCTs/NRSI (18) | no consideration |
| Hong 2019 [48] / Asia | COPD | telemonitoring | control group that didn’t receive telemonitoring | not specified | RCTs (27) | no consideration |
| Jang 2021 [49] / Asia | COPD | telemonitoring | usual care (no telemonitoring intervention) | number of hospitalisations and/or emergency room visits due to COPD exacerbations | RCTs (22) | no consideration |
| Janjua 2021a [50] / Europe | COPD | digital technology interventions with or without routine supported self-management | usual care or control treatment for self-management | (1) Impact on health behaviours (2) Self-efficacy for managing chronic disease (3) Quality of Life (4) Dyspnoea symptoms (5) Exacerbations | RCTs (14) | no consideration |
| Janjua 2021b [51] / Europe | COPD | telehealth intervention without telerehabilitation | usual care | (1) Exacerbations (2) Quality of Life (3) Dyspnoea symptoms (4) Hospital service utilisation (5) Mortality (all-cause) | RCTs (29) | no consideration |
| Kamei 2013 [52] / Asia | COPD | telehome monitoring-based telenursing | usual care | not specified | RCTs/NRSI (7) | no consideration |
| Kruse 2019 [53] / North America | COPD | telemonitoring | not specified | not specified | RCTs/NRSI (29) | no consideration |
| Liu 2020 [54] /Asia | COPD | telemedicine | any kind of comparator | (1) Hospitalisation rate (2) Quality of Life | RCTs (27) | no consideration |
| Lu 2021 [55] / Asia | COPD | telemedicine | any kind of comparator (except other telemedicine intervention) | (1) Emergency room visits (2) Readmissions: exacerbation-related (3) AE-related readmissions, all-cause readmissions, and the rate of AE-related readmissions | RCTs (17) | no consideration |
| Lundel 2015 [56] / Europe | COPD | home-based telehealthcare | any kind of comparator | (1) Physical activity level (2) physical capacity (3) dyspnea | RCTs (9) | no consideration |
| Martinez-Garcia 2017 [57] / Europe | COPD | smartphone devices for promoting physical activity and exercise | not specified | not specified | RCTs/NRSI (8) | no consideration |
| McCabe 2017 [58] / Europe | COPD | computer and mobile technology interventions for self-management | face-to-face and/or hard copy/digital documentary educational/self-management support | (1) Hospital admissions (2) Acute exacerbations requiring general practitioner visit or additional treatment, or both (3) Quality of Life (health-related) | RCTs (3) | Subgroup analysis including age were planned but could not performed, , age as part of suggestions for future research, no consideration of sex or gender |
| McLean 2011 [59] / Europe | COPD | telehealthcare | usual care (face to face) | (1) Total exacerbations (2) Quality of life (3) Emergency Department visits (4) Hospitalisations (5) Deaths | RCTs (10) | Subgroup analysis including age were planned but could not performed, no consideration of sex or gender |
| Michaelchuk 2022 [60] / North America | COPD | home-based telehealth pulmonary rehabilitation | not specified | not specified | RCTs/NRSI (38) | no consideration |
| Polisena 2010 [61] / North America | COPD | home telehealth (telemonitoring or telephone support) | usual care | (1) Quality of Life  (2) health-care resource utilization | RCTs/NRSI (9) | no consideration |
| Sabahi 2021 [62] / Asia | COPD | telemedicine services | not specified | patients’ adherence | RCTs/NRSI (21) | no consideration |
| Shaw 2020 [63] /Europe | COPD | mobile device application interventions for COPD self-management. | usual care | exacerbations | RCTs/NRSI (13) | no consideration |
| Song 2019 [64] / Asia | COPD | home-based telehealth | usual care | (1) 6 min walking distance (2) health status | RCTs (32) | no consideration |
| Sul 2020 [65] / Asia | COPD | telemonitoring | usual care | exacerbation rates | RCTs (28) | a stratified analysis should be conducted according to patient characteristics, including sex and age, in future research |
| Yang 2018 [66] / Asia | COPD | mobile phone application for self-management | usual care | hospital admission | RCTs (8) | no consideration |

Abbreviations:

aCOPD: chronic obstructive pulmonary disease.

bRCTs: randomized controlled trials.

cNRSI: non-randomized studies of interventions.
