# Supplementary material for: Consideration of Sex, Gender, or Age on Outcomes of Digital Technologies for Treatment and Monitoring of Chronic Obstructive Pulmonary Disease: Overview of Systematic Reviews
Source: J Med Internet Res. 2023 Nov 29;25:e49639. doi: 10.2196/49639 (PMC10719824; doi:10.2196/49639)
Supplement: Multimedia Appendix 4 [file jmir_v25i1e49639_app4.docx]

## Figure S1. Overall results of Overlap for Overviews with the GROOVE tool.

| **Overall results** | | |
| --- | --- | --- |
|  |  |  |
| Number of columns (number of reviews) | c | 30 |
| Number of rows (number of index publications) | r | 182 |
| Number of included primary studies (including double counting) | N | 404 |
| Covered area | N/(rc) | 7,40% |
| Corrected covered area | (N-r)/(rc-r) | 4,21% |
| Interpretation of overlap | **Slight overlap** | |
| Structural Zeros | X | 1057 |
| Corrected covered area  (adjusting by structural zeros) | (N-r)/(rc-r-X) | 5,26% |
|  |  |  |
| N° of non-overlapped primary studies | In 1 SR | 108 |
| Number of overlapped primary studies | In 2 SRs | 31 |
|  | In 3 SRs | 12 |
|  | In 4 SRs | 7 |
|  | In 5 SRs | 9 |
|  | In 6 SRs | 4 |
|  | In 7 SRs | 3 |
|  | In 8 SRs | 1 |
|  | In 9 SRs | 3 |
|  | In 10 SRs | 1 |
|  | In 11 SRs | 2 |
|  | In 12 SRs | 0 |
|  | In 13 SRs | 1 |
|  | In 14 SRs | 0 |
|  | In 15 or more SRs | 0 |

SR = systematic review
